# Unusual Bullous Manifestation of Scabies in Childhood Diagnosed by Dermoscopy: A Case Report

**DOI:** 10.1002/ccr3.73030

**Published:** 2026-06-23

**Authors:** Anupa Khadka, Vikash Paudel

**Affiliations:** ^1^ Patan Academy of Health Sciences Lalitpur Nepal

**Keywords:** bullous scabies, dermoscopy, pediatrics, scabies

## Abstract

Scabies is a common ectoparasitic infestation, but atypical variants such as bullous scabies are rare and often misdiagnosed. We report a 12‐year‐old boy who presented with pruritic lesions comprising predominantly vesicles and bullae over the hands, along with a few scattered excoriated papules on the trunk and thighs. Dermoscopic evaluation revealed the characteristic “jet with contrail” sign in the interdigital area, confirming the diagnosis of scabies according to the 2020 International Alliance for the Control of Scabies criteria. The patient was treated with two applications of topical 5% permethrin 1 week apart, along with symptomatic therapy, resulting in complete resolution without sequelae at 4 weeks. This case highlights bullous scabies as an underrecognized pediatric presentation and underscores the value of dermoscopy as a rapid, noninvasive diagnostic tool that can prevent misdiagnosis, avoid unnecessary investigations, and ensure timely, effective treatment.

## Key Clinical Message

Bullous scabies in children is an underrecognized presentation that frequently mimics other bullous dermatoses, leading to diagnostic delays and inappropriate treatment. Clinicians must maintain a high index of suspicion for scabies in pediatric patients presenting with atypical bullous eruptions to ensure timely and effective management.

## 1 Introduction

Scabies is a common parasitic infestation caused by *Sarcoptes scabiei var. hominis*, affecting more than 400 million individuals each year. Despite its high prevalence, the World Health Organization classified scabies as a Neglected Tropical Disease in 2017, with the children in resource‐limited settings bearing a disproportionate burden, accounting for approximately 5%–50% of cases [1]. While classical scabies is readily identifiable by characteristic features such as burrows, papulo‐vesicles, nodules, and excoriations, rare atypical presentations, including the bullous variant, have been reported. Early recognition of such atypical variants is crucial to prevent ongoing transmission, avoid misdiagnosis, and reduce unnecessary diagnostic investigations and treatment costs.

## 2 Case History/Examination

**FIGURE 1:**
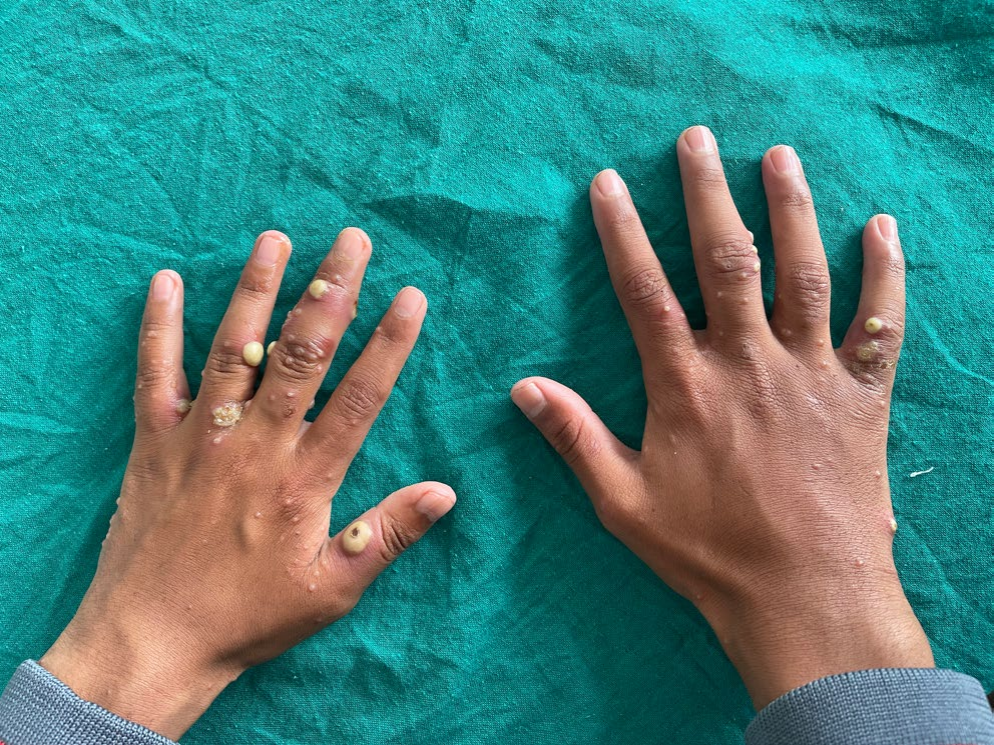
Multiple clear fluid‐filled vesicles and turbid fluid‐filled bullae over the dorsum of the hands. A few papules are also noted occasionally.

**FIGURE 2:**
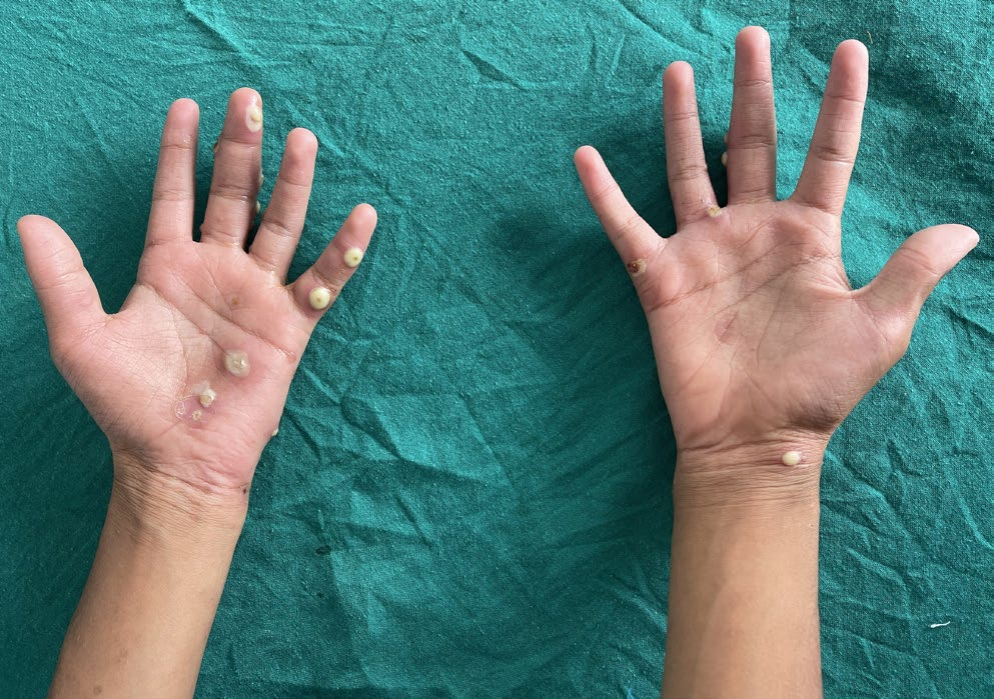
Vesico‐bullous lesions involving the palms.

A twelve‐year‐old boy presented with severely itchy lesions over the bilateral hands, abdomen, and thighs for a duration of 2 weeks. Pruritus was predominantly nocturnal. Similar symptoms and lesions in the close contact were absent. Cutaneous examination revealed multiple, a few millimeters to about 1 centimeter in diameter, clear to turbid fluid‐filled vesicles and bullae distributed over the dorsal and palmar aspects of bilateral hands. A few areas of crusted erosion and desquamation of resolving lesions were noted on the hands (Figures 1 and 2). A few excoriated erythematous papules over the lower abdomen and medial thighs were also noted, while the genitalia were uninvolved.

## 3 Differential Diagnosis, Investigations, and Treatment

**FIGURE 3:**
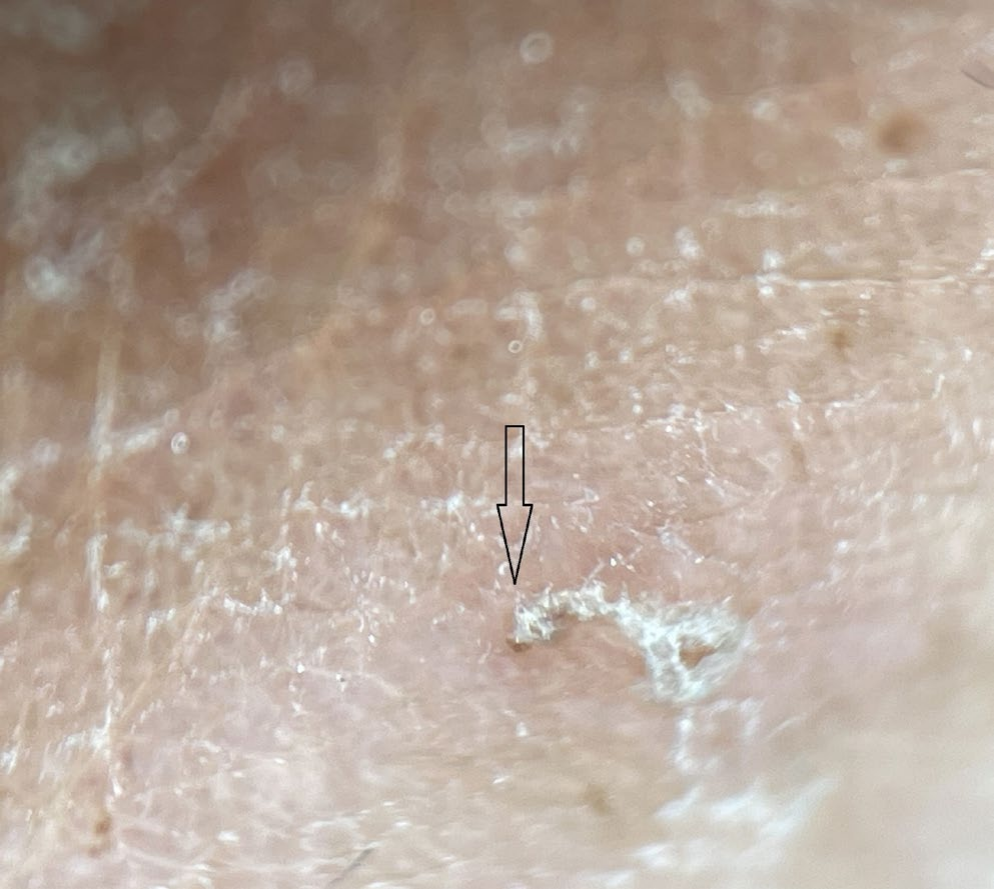
Dermoscopy (Polarized 20 X): Mite and burrow presenting a “Jet with contrail or Delta wing” sign.

Based on clinical presentation, a differential diagnosis of arthropod bite reaction, bullous impetigo, acute contact dermatitis, bullous pemphigoid, and classical scabies with secondary bacterial infections was considered. Culture from the lesion showed no growth. Skin scraping failed to reveal mites in saline mount. However, dermoscopy revealed the typical “Jet with contrail” sign in the finger web area (Figure 3), consistent with scabies. A diagnosis of Bullous scabies was made in accordance with the *2020 International Alliance for the Control of Scabies Consensus Criteria for the Diagnosis of Scabies* as Confirmed scabies (level A3) [2]. Histopathology and direct immunofluorescence were not performed as the diagnosis was confidently established on clinical and dermoscopic grounds. In the resource‐limited Nepalese setting, these investigations would incur additional costs and delay. Additionally, the caregivers were unwilling to consent to an invasive test once a non‐invasive diagnosis had been made. The patient was treated with 5% permethrin lotion, applied topically in two doses 1 week apart, along with oral antihistamines for symptomatic relief of pruritus. Appropriate counseling regarding application technique, hygiene measures, treatment of close contacts, and follow‐up was provided.

## 4 Outcome and Follow‐Up

Complete resolution of the lesions was observed, with no residual scar or pigmentation, at 4 weeks of follow‐up.

## 5 Discussion

Scabies is a prevalent dermatological condition in Nepal, with prevalence reported from 5.15% in urban tertiary hospital patients to 18.36% in rural populations presenting to regional hospital; however, bullous scabies has not yet been documented locally [3].

In most instances, the diagnosis of classical scabies is clinically straightforward. However, atypical variants, including crusted scabies, nodular scabies, scabies incognito, nail scabies, and bullous scabies, have been described [4, 5]. Bullous scabies is a rare manifestation of scabies, predominantly reported in older males, with only sporadic cases documented in the pediatric population [6, 7, 8, 9]. While burrows and male genital lesions are highly specific for scabies, vesicles on the palms and soles are considered “typical lesions” only in infants [2]. Most cases report bullae and vesicles occurring alongside classical scabietic lesions; however, in our case, vesicles and bullae were the predominant presenting features [8, 9]. Bullae in scabies are predominantly reported in the extremities—the thighs and arms, with palmar involvement still mainly reported in infants and younger children [6].

Although the exact pathogenesis remains unclear, several mechanisms have been proposed for bulla formation in scabies, including scabies‐induced immune response, auto‐eczematization, direct epidermal injury, or the secretion of lytic enzymes by the mites, and immunologic cross‐reactivity between scabies antigens and basement membrane zone (BMZ) proteins [6, 7, 8]. Additionally, Arslan et al. reported *Staphylococcus* or *Streptococcus* infection in five pediatric cases, supporting the role of secondary bacterial infection in the pathogenesis of pediatric bullous scabies [6].

Dermoscopy and skin scraping can be used to ascertain a definitive diagnosis even in atypical forms. Dermoscopy demonstrates high diagnostic accuracy, with reported sensitivity and specificity of 98.3% and 88.5%, respectively [10]. Compared with skin scraping, dermoscopy is reported to be more sensitive and offers the advantages of reduced invasiveness and shorter examination time than other investigations [6, 10]. Histopathology findings of scabietic bullae have been reported to be mainly subepidermal and occasionally intraepidermal cleavage with mixed inflammatory infiltrates, predominantly comprising neutrophils and eosinophils. Direct immunofluorescence (DIF) is reported to be positive (mainly linear deposits of IgG and C3 in BMZ) in many cases. In contrast, indirect immunofluorescence (IIF) reports circulating IgG to BMZ components only in a few cases [6, 8, 11, 12]. These findings closely resemble those observed in bullous pemphigoid, which may lead to diagnostic ambiguity regarding association, causality, or sequelae. Owing to overlapping clinical and immunopathological features, bullous scabies is frequently misdiagnosed and treated as bullous pemphigoid. Further complicating this distinction, reports are suggesting the subsequent development of true bullous pemphigoid following bullous scabies, supporting a possible pathogenic or trigger relationship [11, 12, 13].

Recent advances in scabies diagnostics, including reflectance confocal microscopy (RCM), line‐field confocal optical coherence tomography (LC‐OCT), and PCR‐based assays, have enhanced diagnostic accuracy through high‐resolution visualization of mites and sensitive molecular detection of mite DNA, respectively. However, their use remains limited in endemic and resource‐constrained settings due to cost, technical requirements, and limited accessibility, leaving diagnosis largely dependent on clinical evaluation and conventional microscopy [5].

In such instances, direct methods for mite identification or empiric anti‐scabicidal therapy guided by clinical suspicion must be adopted, as in our case. Importantly, the therapeutic approach to bullous scabies does not differ from that of classical scabies, and standard topical or oral anti‐scabicidal regimens are generally sufficient for effective management.

Although precise epidemiological data are limited, scabies remains a common infestation with diverse clinical variants. The bullous form is rare and frequently misdiagnosed or disregarded, which may result in inappropriate treatment, continued transmission, iatrogenic spread, and complications such as post‐streptococcal glomerulonephritis. Maintaining a high index of suspicion, particularly in endemic areas, is crucial for early diagnosis and appropriate management, as this condition may be more common than currently reported.

## Author Contributions

**Anupa Khadka:** conceptualization, writing – original draft, writing – review and editing. **Vikash Paudel:** writing – review and editing.

## Funding

The authors have nothing to report.

## Ethics Statement

The authors have nothing to report.

## Consent

Written informed consent was obtained from the patient for publication of this case report and any accompanying images.

## Conflicts of Interest

The authors declare no conflicts of interest.

## Data Availability

No datasets were generated or analyzed beyond the patient's clinical records, laboratory reports, and biopsy findings. All relevant information is included within the article.
